# Practical Diagnosis

**Published:** 1898-09

**Authors:** 


**Practical Diagnosis.** By HOBART AMORY HARE, M.D. Second Edition. Pp. xii., 17—605. London: Henry Kimpton. 1897.

The first edition of this book, published in August, 1896, was rapidly exhausted, and hence the necessity for this one, which has been revised and enlarged. Its object is to place before the physician and the student the subject of medical diagnosis as it is met at the bedside, and to this end the symptoms used in diagnosis are discussed first and their application to determine the character of the disease follows. It will he seen that this method is the reverse of that adopted in the ordinary text-books on medicine. Most of the 201 engravings and 13 coloured plates with which the book is enriched are modern and good. The tabetic foot (page 118) and tabetic ulcer (page 126) are fairly representative of the original photographs.

The introductory chapter on general diagnostic considerations inculcates the necessity for keen observation of the patient as he walks into the consulting-room, or of his surroundings when he is visited at home. The clothing, the gait, the build, the voice, the expression, and the manner, are all likely to give much information, which is at once assimilated almost unconsciously by the experienced physician, who will often make a diagnosis whilst observing the patient from perhaps the foot of the bed and before any answers have been given or questions put.

Part I. commences with a chapter on the face and head; it is a very interesting one, the different varieties of expression are well described, the movements and position of the head and neck and the various indications of facial paralysis and spasm are indicated, and this chapter has numerous good illustrations, such as the mouth-breather, general anasarca, cretinism, acromegaly, myxœdema, ptosis, and goitre.

The descriptions of the manifestations of disease in the hands and arms and feet and legs have some excellent drawings illustrating the deformities of gout, and some Röntgen ray pictures showing the condition of the bones and the contrast between gout and chronic rheumatoid arthritis. The phenomena of muscular wasting and athetosis are also well depicted. A short chapter on hemiplegia gives a very complete pictorial demonstration of its etiology and clinical features. Succeeding chapters comprise the clinical aspects of the tongue, mouth and pharynx, the eye, the skin, the thorax and its viscera, the abdomen and the abdominal viscera, and this is followed by an outline of the changes in the blood in which the use of the hæmatocrit attachment to the ordinary centrifugal machine is advocated as a ready method for ascertaining the number and the quality of the corpuscles. The differences between the blood of pernicious anæmia and chlorosis are pointed out, and some good drawings copied from Rieder and Osler show the features of the Myelocyte, the Normoblast, and the Megaloblast. It is scarcely yet sufficiently realised that examination of the blood is not so difficult as is commonly supposed, but that "about all that is necessary to be known about the blood in a given case can be ascertained in fifteen minutes. To make a thorough examination of blood, it is necessary to find out the following facts: Number of red cells in a given quantity, which is usually a centimetre; number of white cells in the same; ratio of white to red; number of each kind of white cells; ratio to each other; size, shapes, peculiarities, and contents of cells; percentage of hæmoglobin; color value or relative amount of hæmoglobin per red cell; relative amount of fibrin; and finally, if any substance other than the above occurs, and if so, of what nature." (*Medical Record*, Nov. 6, 1897, p. 663.) The different steps by which this information is to be obtained are briefly indicated, and an outline of the recognised parasites of blood follows, including the varieties of the malarial forms of Laveran and the filaria sanguinis hominis.

The utility of the Widal test in doubtful cases of enteric fever is commented upon. This test has now become well established, and we ought to hear nothing more in the future as to the difficulties in diagnosis between influenza and typhoid fever. The possibilities of diagnosis founded upon the presence and degree of leucocytosis open up various questions for future and further investigation, as, for instance, the differentiation of pericarditis with effusion from hypertrophy or dilatation of the heart. The following chapters, on the urinary bladder and the urine, and the bowels and fæces, contain much useful information imparted in an original way.

Part II. is made up of nine chapters on such things as fever and sub-normal temperatures, headache and vertigo, coma, convulsions, vomiting, cough, pain, tendon-reflexes, and speech. An excellent index makes this well-printed and excellently illustrated volume very convenient for easy reference to any point.

